# Supplementary material for: Examining Cultural Differences in the Associations between Appraisals and Emotion Regulation and PostTraumatic Stress Disorder in Malaysian and Australian Trauma Survivors
Source: Int J Environ Res Public Health. 2022 Jan 21;19(3):1163. doi: 10.3390/ijerph19031163 (PMC8834521; doi:10.3390/ijerph19031163)
Supplement: Supplementary file 1 [file ijerph-19-01163-s001.zip › ijerph-1525500-supplementary.pdf]

Supplemental Table S1

Correlation analyses for each cultural group separately

	Australian Group<br>PTSD Symptoms	Malaysian Group<br>PTSD Symptoms
Self-Blame	.57**	.23*
Negative Self	.67**	.58**
Negative World	.57**	.51**
PTCI Total	.68**	.56**
Primary Control	.01	.26*
Secondary Control	-.17	-.11
Fatalism	.11	.09
Chinese Beliefs about Adversity	-.25*	-.18*
Trauma-Specific Rumination	.62**	.65**
Rumination - Brooding	.42**	.59**
Rumination - Reappraisal	.38**	.44**
Reappraisal	.003	-.17
Suppression	.25*	.35*
Enhance positivity	-.14	-.24*
Perspective taking	.06	-.04
Soothing	-.06	-.09
Social modelling	-.08	-.10
IERQ total	-.07	-.14

*$p < .05$; ** $p < .01$. *Note.* PTCI= Posttraumatic Cognitions Inventory, IERQ= Interpersonal Emotion Regulation Questionnaire. PTSD = Posttraumatic stress disorder.

Supplemental Table S2

Summary of Moderation Results with Posttraumatic Stress Disorder Symptoms as the Outcome Variable.

	b [95% CI]	*SE*	*t*
**Self-Blame** R^2=.23, F (7, 203)= 8.42**			
Constant	40.71 [24.56-56.86]	8.19	4.97***
Self-Blame	-.48 [-1.37-.40]	.45	1.07
Cultural Group	-19.12 [-29.28—8.97]	5.15	3.71***
Self-Blame x Cultural Group	.98 [.39-1.56]	.30	3.29**
**Negative Self** R^2=.42, F (7, 203)= 21.01***			
Constant	15.16 [-.23-30.56]	7.81	1.94
Negative Self	.31 [.08-.54]	.12	2.65**
Cultural Group	-7.88 [-.17.12-1.35]	4.86	1.68
Negative Self x Cultural Group	.07 [-.07-.22]	.07	.99
**Negative World** R^2=.32, F (7, 203)= 13.43***			
Constant	16.55 [-3.06-36.16]	9.95	1.66
Negative World	.60 [.004-1.19]	.30	1.99*
Cultural Group	-9.24 [-20.95-2.46]	5.94	-1.56
Negative World x Cultural Group	.19 [-.19-.57]	.19	1.00
**Fatalism** R^2=.04, F (7, 203)= 1.32			
Constant	24.60 [-10.66-59.85]	17.88	1.38
Fatalism	.53 [-1.18-2.24]	.87	.61
Cultural Group	-1.56 [-24.03-20.91]	11.40	.14
Fatalism x Cultural Group	-.18 [-1.24-.89]	.54	.33
**Beliefs Adversity** R^2=.05, F (7, 203)= 1.60			
Constant	52.04 [.71-103.38]	26.04	2.00*
Beliefs Adversity	-.58 [-1.94-.78]	.69	.84

Cultural Group	-.90 [-32.70-30.90]	16.13	.06
Beliefs Adversity x Cultural Group	-.04 [-.87-.79]	.42	.09
**Reappraisal** R^2=.05, F (7, 203)= 1.64			
Constant	14.20 [-21.32-49.73]	18.02	.79
Reappraisal	.66 [-.59-1.91]	.63	1.04
Cultural Group	14.15 [-10.53-38.82]	12.52	1.13
Reappraisal x Cultural Group	-.61 [-1.44-.21]	.42	1.46
**Suppression** R^2=.13, F (7, 203)= 4.34***			
Constant	29.79 [2.85-56.73]	13.66	2.18*
Suppression	.38 [-1.11-1.87]	.76	.50
Cultural Group	-14.62 [-31.55-2.30]	8.59	1.70
Suppression x Cultural Group	.48 [-.47-1.44]	.48	.99
**Enhancing Positivity** R^2=.08, F (7, 203)= 2.47*			
Constant	41.67 [5.29-78.05]	18.45	2.26*
Enhancing Positivity	-.30 [-2.11-1.52]	.92	.32
Cultural Group	.45 [-20.49-21.40]	10.62	.04
Enhancing Positivity x Cultural Group	-.31 [-1.39-.78]	.55	.56
**Perspective Taking** R^2=.04, F (7, 203)= 1.28			
Constant	26.66 [5.41-47.91]	10.78	2.47*
Perspective taking	.68 [-.87-2.23]	.79	.86
Cultural Group	.70 [-13.74-15.14]	7.32	.10
Perspective Taking x Cultural Group	-.42 [-1.41-.57]	.50	-.84
**Soothing** R^2=.05, F (7, 203)= 1.38			
Constant	33.48 [9.86-57.10]	11.98	2.79**
Soothing	.06 [-1.44-1.56]	.76	.08
Cultural Group	-1.11 [-16.06-13.85]	7.58	.15
Soothing x Cultural Group	-.21 [-1.15-.72]	.47	.45
**Social Modelling** R^2=.05, F (7, 203)= 1.49			

Constant	34.55 [8.33-60.77]	13.30	2.60*
Social Modelling	-.07 [-1.67-1.54]	.81	.08
Cultural Group	-.74 [-17.72-16.24]	8.61	.09
Social Modelling x Cultural Group	-.20 [-1.21-.82]	.51	.38

*p < .05; ** p < .01; ***p < .001.

Exploratory Analyses – Moderated Mediation Analyses

PTCI

Interdependent, effect= .03, *SE*= .03, 95% CI[-.01, .09], and independent self-construal, effect= .01, *SE*= .03, 95% CI[-.05, .07], did not mediate the relationships between self-blame and PTSD symptoms. Interdependent, effect= .002, *SE*= .01, 95% CI[-.01, .01], and independent self-construal, effect= -.001, *SE*= .01, 95% CI[-.02, .02], did not mediate the relationships between negative self and PTSD symptoms. Interdependent, effect= .01, *SE*= .02, 95% CI[-.02, .04], and independent self-construal, effect= .01, *SE*= .02, 95% CI[-.02, .04], did not mediate the relationships between negative world and PTSD symptoms.

Fatalism and Chinese Cultural Beliefs about Adversity

Independent self-construal mediated the association between fatalism appraisals and PTSD symptoms, effect= -.12, *SE*= .07, 95% CI[-.28, -.01]. There was no evidence of cultural group moderating this conditional indirect effect, index= -.16, SE= .11, 95% CI[-.42, .01]. Interdependent self-construal did not mediate the relationship between fatalism and PTSD symptoms, effect= .17, *SE*= .10, 95% CI[-.002, .38]. There was no evidence for cultural group moderating this mediation effect, index= 0.01, SE= .08, 95% CI[-.18, .16]. Interdependent self-construal mediated the association between Chinese cultural beliefs about adversity appraisals and PTSD symptoms, effect= .14, *SE*= .06, 95% CI[.04, .28]. There was no evidence of cultural group moderating this conditional indirect effect, index= -.05, SE= .08, 95% CI[-.22, .12]. Independent self-construal did not mediate the relationship between cultural beliefs about adversity and PTSD symptoms, effect= -.08, *SE*= .13, 95% CI[-.33, .17]. There was no evidence of cultural group moderating this conditional indirect effect, index= -.01, SE= .04, 95% CI[-.11, .07].

Reappraisal and Suppression

Interdependent self-construal did mediate the relationship between reappraisal and PTSD symptoms, effect= .14, SE= .07, 95% CI[.02, .28]. There was no evidence of cultural group moderating this indirect effect, index= .03, SE= .08, 95% CI[-.13, .20]. Independent self-construal did not significantly mediate the relationships between reappraisal and PTSD symptoms, effect= -.14, SE= .08, 95% CI[-.30, .02]. There was no evidence of cultural group moderating this indirect effect, index= -.11, SE= .09, 95% CI[-.32, .03]. Interdependent, effect= .06, SE= .05, 95% CI[-.02, .17], and independent, effect= .04, SE= .05, 95% CI[-.04, .15], self-construal did not mediate the relationships between suppression and PTSD symptoms. There was no evidence of cultural group moderating these indirect effects, independent index= -.10, SE= .09, 95% CI[-.30, .05], interdependent index= .01, SE= .06, 95% CI[-.11, .16].

Interpersonal Emotion Regulation

Enhanced positivity. Interdependent self-construal mediated the relationships between enhanced positivity and PTSD symptoms effect= .23, SE= .08, 95% CI[.07, .39]. There was no evidence for cultural group moderating this mediation, index= 0.03, SE= .13, 95% CI[-.22, .30]. Independent self-construal did not mediate the relationships between enhanced positivity and PTSD symptoms, effect= -.12, SE= .08, 95% CI[-.29, .02]. There was no evidence for cultural group moderating this mediation, index= -.06, SE= .10, 95% CI[-.30, .10].

Perspective taking. Interdependent self-construal mediated the relationships between perspective taking and PTSD symptoms effect= .19, SE= .09, 95% CI[.01, .38]. There was no evidence of cultural group moderating this conditional indirect effect, index= .13, SE= .10, 95% CI[-.03, .36]. Independent self-construal also mediated the relationships between perspective taking and PTSD symptoms effect= -.15, SE= .08, 95% CI[-.32, -.01].

There was no evidence of cultural group moderating this conditional indirect effect, index= -.10, SE= .11, 95% CI[-.38, .06].

Soothing. There was evidence of cultural group differences between the conditional indirect effects, index= .24, SE= .13, 95% CI[.02, .52]. Interdependent self-construal mediated the relationships between soothing and PTSD symptoms for the Malaysian group, effect= .18, SE= .09, 95% CI[.01, .38], but not the Australian group, effect= -.06, SE= .06, 95% CI[-.21, .05]. There was no evidence of cultural group moderating the conditional indirect effect for independent self-construal, index= -.09, SE= .07, 95% CI[-.24, .04]. Independent self-construal did not mediate the relationships between soothing and PTSD symptoms effect= -.14, *SE*= .08, 95% CI[-.32, .001].

Social Modelling. There was evidence of cultural group moderating the conditional indirect effects for interdependent self-construal, index= .22, SE= .13, 95% CI[.01, .51]. Interdependent self-construal mediated the relationships between social modelling and PTSD symptoms for the Malaysian group, effect= .24, SE= .16, 95% CI[.04, .49], but not the Australian group, effect= .02, SE= .09, 95% CI[-.13, .23]. Independent self-construal did not mediate the relationships between soothing and PTSD symptoms effect= -.13, SE= .08, 95% CI[-.30, .06]. There was no evidence of cultural group moderating the conditional indirect effect, index= -.09, SE= .11, 95% CI[-.36, .06].
